# *CYP1A1* Ile462Val Polymorphism Is Associated with Cervical Cancer Risk in Caucasians Not Asians: A Meta-Analysis

**DOI:** 10.3389/fphys.2017.01081

**Published:** 2017-12-18

**Authors:** Li-Na Wang, Fen Wang, Jie Liu, Ying-Hui Jin, Cheng Fang, Xue-Qun Ren

**Affiliations:** ^1^Department of Gynaecology, Huaihe Hospital of Henan University, Kaifeng, China; ^2^Center for Evidence-Based Medicine, Henan University, Kaifeng, China; ^3^Department of Gynaecology and Obstetrics, The First Affiliated Hospital of Nanchang University, Nanchang, China; ^4^Department of General Surgery, Huaihe Hospital of Henan University, Kaifeng, China; ^5^Center for Evidence-Based and Translation Medicine, Zhongnan Hospital of Wuhan University, Wuhan, China; ^6^Department of Evidence-Based Medicine and Clinical Epidemiology, The Second Clinical College, Wuhan University, Wuhan, China

**Keywords:** *CYP1A1*, Ile462Val, cervical cancer, polymorphism, risk

## Abstract

**Objective:** Previous studies have reported that Ile462Val polymorphism in the gene Cytochrome P450 1A1 (*CYP1A1*) is associated with the risk of cervical cancer, but inconsistent results have emerged. Hence, we performed this updated and cumulative meta-analysis to ascertain a more accurate association between *CYP1A1* Ile462Val polymorphism and risk of cervical cancer.

**Methods:** Studies involving the *CYP1A1* Ile462Val polymorphism associated with cervical cancer risk were searched from the databases of PubMed, Scopus, ScienceDirect, and Chinese National Knowledge Infrastructure (CNKI). The strength of correlation was evaluated through calculating summary odds ratios (ORs) with the corresponding 95% confidence intervals (95% CIs). Subgroup analyses according to ethnicity, source of control and HWE were completed to further explore specific association between the polymorphism and the cancer risk.

**Results:** Altogether, 11 eligible case-control studies were ultimately encompassed into the current meta-analysis, with 1,932 patients and 2,039 healthy controls. The total analysis revealed a borderline relationship between *CYP1A1* Ile462Val polymorphism and cervical cancer risk in general population. Interestingly, after subgroup analyses based on ethnicity and source of control, the polymorphism increased the susceptibility of cervical cancer in Caucasian (G vs. A: OR = 1.97, 95% CI = 1.24–3.13; GG vs. AA: OR = 3 .24, 95% CI = 1.24–8.46; GA vs. AA: OR = 1.62, 95% CI = 1.25–2.10; GA+GG vs. AA: OR = 1.68, 95% CI = 1.16–2.43; GG vs. AA+GA: OR = 2.73, 95% CI = 1.05–7.10) and population-based (G vs. A: OR = 1.49, 95% CI = 1.10–2.02; GA vs. AA: OR = 1.41, 95% CI = 1.20–1.67; GA+GG vs. AA: OR = 1.40, 95% CI = 1.19–1.64) groups.

**Conclusion:** The *CYP1A1* Ile462Val polymorphism may enhance the susceptibility to cervical cancer in Caucasian females.

## Introduction

Cervical cancer is followed by breast cancer, which is the first common cancer among women all over the world (Denslow et al., 2012). Besides the onset of cervical cancer was aged 15–49 years in developing countries (Forouzanfar et al., 2011). Cervical cancer is a major threat to woman's health and quality of life, and is also the focal point and the difficulties for medical workers. Hence, to seek the risk factor and people who may be at high risk of cervical cancer for prevention is a significant and important work. As we know, smoking (Sood, 1991; Zeng et al., 2012) and human papillomavirus (HPV) infection (Patel et al., 2016) are the classical risk factor for cervical cancer. However, some women without smoking and HPV infection also got cervical cancer, why? That's might be other factors play a role in the onset of cervical cancer, such as genetic background.

Cytochrome P450 (CYP) gene family and susceptibility to many cancers has been the most widely studied (Rodriguez-Antona et al., 2010). *CYP1A1* is belonged to CYP gene family1, subfamily A, polypeptide 1 and has two major functional nonsynonymous polymorphisms: MspI polymorphism m1 and Ile462Val polymorphism m2 (Sugawara et al., 2003). Ile462Val polymorphism is a heme-binding site due to the replacement of isoleucine (Ile) by valine (Val), which caused by G to A transition (A4889G) in exon 7 at codon 462, also called rs1048943 (https://www.ncbi.nlm.nih.gov/projects/SNP/snp_ref.cgi?rs=1048943). There are five published meta-analyses directly or indirectly investigated the association between *CYP1A1* Ile462Val polymorphism and risk of cervical cancer (Sergentanis et al., 2012; Yang et al., 2012; Wu et al., 2013; Qin et al., 2014; Wang et al., 2015), however, they obtained inconsistent results and with some deficiency (Table 1). For example, the meta-analysis performed by Yang et al. was conducted in 2012 involving ten case-control studies, and found that *CYP1A1* Ile462Val polymorphism was associated with increased risk of cervical cancer in general populations. Besides, ethnic subgroup analyses showed a significant association was found in Caucasians but not in Asians (Yang et al., 2012). However, two publications (Geng et al., 2010; Shi et al., 2011) involved the same subjects were both included in above meta-analysis. Moreover, several new original studies (Abbas et al., 2014; Roszak et al., 2014; Li et al., 2016) on this topic were published since then. The recent meta-analysis by Wang et al. pooled the data of 8 case-control studies and indicated the *CYP1A1* Ile462Val polymorphism might be a risk factor for cervical cancer (Wang et al., 2015). Since data extracted from two articles (Sugawara et al., 2003; Joseph et al., 2006) were repeatedly included in the meta-analysis, six case-control studies were actually included. Also, due to different inclusion criteria and uneven sample sizes, several meta-analyses (Wu et al., 2013; Qin et al., 2014) presented opposite conclusions. Additionally, subgroup analyses by ethnicity could not be performed due to limited number of studies.

**Table 1:** Characteristics of previous published meta-analyses on *CYP1A1* Ile462Val polymorphism and cervical cancer risk.

**References**	**Search databases**	**End of search**	**Included studies**	**Study number**	**Topic**
Sergentanis et al., 2012	MEDLINE	October, 2010	Sugawara et al., 2003; Joseph et al., 2006; Taskiran et al., 2006; Gutman et al., 2009	4 case–control studies	MspI and Ile462Val polymorphisms and cervical cancer risk
Yang et al., 2012	PubMed, Embase, and CBM	May, 2012	Sugawara et al., 2003; Huang et al., 2006; Joseph et al., 2006; Taskiran et al., 2006; Gutman et al., 2009; Zhang, 2009, 2011; Geng et al., 2010; Ding et al., 2011; Shi et al., 2011	10 case–control studies	*CYP1A1* Ile462Val polymorphism and cervical cancer risk
Wu et al., 2013	PubMed	December 31, 2012	Sugawara et al., 2003; Joseph et al., 2006; Gutman et al., 2009	3 case–control studies	MspI and Ile462Val polymorphisms and overall cancer risk
Qin et al., 2014	PubMed, ISI, and EMBASE	April 15, 2013	Sugawara et al., 2003; Joseph et al., 2006; Taskiran et al., 2006; Gutman et al., 2009	4 case–control studies	*CYP1A1* Ile462Val polymorphism and cancer risk
Wang et al., 2015	PubMed and Embase	June, 2014	Sugawara et al., 2003; Joseph et al., 2006; Taskiran et al., 2006; Gutman et al., 2009; Abbas et al., 2014; Roszak et al., 2014	8 case–control studies	42 SNPs with genetic risk for cervical cancer

Thus, we identified relevant published reports through a systematic search strategy, and performed this updated and cumulative meta-analysis to reappraise between *CYP1A1* Ile462Val polymorphism and risk of cervical cancer. What's more, subgroup and sensitivity analyses were conducted to further ascertain such relationship.

## Materials and methods

### Ligibility criteria

Any study was considered eligible if it met all of the following criteria: (1) contain information investigated the association between *CYP1A1* Ile462Val polymorphism and risk of cervical cancer; (2) as a cohort or case-control design; (3) both cases and controls were clearly diagnosed and all controls were healthy subjects or tissues; (4) included information that allowed for calculation of the odds ratios (ORs) and 95% confidence intervals (CIs). The study investigated the cervical dysplasia, cervical intraepithelial neoplasia (CIN), or cervical squamous intraepithelial lesions (CSIL) was excluded. If any publication involved the same subjects, we compared them according to the population, exposure, control, and study period and chosen the more comprehensive one.

### Literature search

The PubMed, Scopus, ScienceDirect, and Chinese National Knowledge Infrastructure (CNKI) databases were searched for relevant studies that were published up to January 10, 2017. The search strategy included usage of the following key words: cervical, cervix, cancer, carcinoma, *CYP1A1*, cytochrome P-450, cytochrome P450, polymorphism, Ile462Val, A2455G, and rs1048943. Moreover, we also searched through the references which were cited in the included studies to obtain additional relevant studies. Two independent authors searched and evaluated the eligibility of all studies and any disagreements were resolved by discussion.

### Data extraction

Two authors extracted the following data independently from all included studies: the last name of first author, year of publication, country of origin, ethnicity, sources of controls, sample sizes of cases and controls, genotype distribution in cases and controls, Hardy-Weinberg Equilibrium (HWE) for controls, and genotyping methods. If the HWE was not reported, we assessed it by the chi-square test and significance was set at *P* < 0.05 according to the genotype distribution in control. Disagreements were resolved by discussion.

### Data analysis

All analyses were conducted using the Comprehensive Meta-Analysis v2.2 software (Zeng et al., 2016a,b). First, the heterogeneity was evaluated using the *I*^2^ test and Cochran *Q* test, both *I*^2^ < 50% and *P* > 0.1 was considered acceptance heterogeneity and the fixed-effect model was chosen to pool single study; otherwise, the random-effect model was used. Subgroup analyses according to ethnicity, source of control, and HWE were conducted. Five genetic models were used for overall and subgroup analyses: G vs. A, GG vs. AA, GA vs. AA, (GA+GG) vs. AA, and GG vs. (AA+GA). The odds ratio (OR) and its 95% confidence interval (95%CI) were used to evaluate the pooled effect size. Sensitivity analysis was performed to investigate the influence of a single study on overall analysis, and to test the robustness of overall results. Cumulative meta-analysis was carried out to observe the change when with sample sizes were enlarged (Pabalan, 2010; Rotondi and Bull, 2012). Publication bias was assessed by visual inspection of funnel plots and Egger's test.

## Results

### Study selection and characteristics

**Figure 1:**
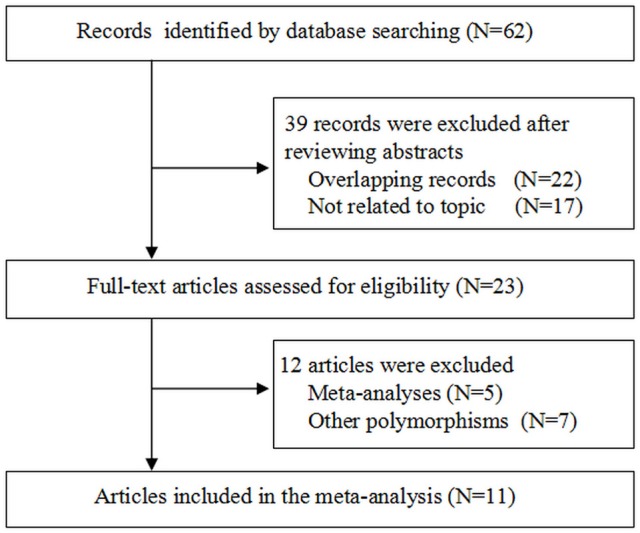
Flow chart of study selection.

Figure 1 shows the study selection process. The primary search yielded potentially related publications and finally 11 case-control studies involving 1,932 patients and 2,039 healthy controls were included (Sugawara et al., 2003; Huang et al., 2006; Joseph et al., 2006; Gutman et al., 2009; Zhang, 2009, 2011; Ding et al., 2011; Shi et al., 2011; Abbas et al., 2014; Roszak et al., 2014; Li et al., 2016). Of these 11 case-control studies, four dealt with probands of Caucasian origin (Joseph et al., 2006; Gutman et al., 2009; Abbas et al., 2014; Roszak et al., 2014) and seven referred to Asian origin (Sugawara et al., 2003; Huang et al., 2006; Zhang, 2009, 2011; Ding et al., 2011; Shi et al., 2011; Li et al., 2016); five studies were out of Hardy Weinberg Equilibrium (HWE) (Huang et al., 2006; Zhang, 2009; Ding et al., 2011; Shi et al., 2011; Abbas et al., 2014). Table 2 lists the main characteristics of identified studies.

**Table 2:** Main characteristics of studies included in this meta-analysis.

**References**	**Country (Ethnicity)**	**Sample size**		**Genotype distribution (Case)**					**Genotype distribution (Control)**					**Source of control**	**Genotyping method**	**HWE**
		**Cases**	**Controls**	**AA**	**AG**	**GG**	**A**	**G**	**AA**	**AG**	**GG**	**A**	**G**			
Sugawara et al., 2003	Japan (Asian)	75	31	48	27	0	123	27	21	10	0	52	10	PB	PCR	Yes
Huang et al., 2006	China (Asian)	113	113	36	62	1	134	64	14	95	1	123	97	HB	PCR-RFLP	No
Joseph et al., 2006	India (Caucasian)	147	165	91	45	11	227	67	136	26	3	298	32	PB	PCR-RFLP	Yes
Gutman et al., 2009	Israel (Caucasian)	43	123	29	14	0	72	14	85	31	5	201	41	PB	PCR-RFLP	Yes
Zhang, 2009	China (Asian)	50	30	14	26	10	54	34	17	7	6	41	19	HB	PCR-RFLP	No
Shi et al., 2011	China (Asian)	176	112	34	121	21	189	163	32	76	4	140	84	PB	PCR-RFLP	No
Ding et al., 2011	China (Asian)	280	280	72	129	79	273	287	83	100	97	266	294	PB	PCR-RFLP	No
Zhang, 2011	China (Asian)	32	114	14	13	3	41	19	34	51	17	119	85	HB	PCR-RFLP	Yes
Abbas et al., 2014	India (Caucasian)	200	208	110	84	6	304	96	114	65	2	347	69	PB	PCR-RFLP	No
Roszak et al., 2014	Poland (Caucasian)	456	495	415	41	0	871	82	466	29	0	961	29	PB	PCR-RFLP	Yes
Li et al., 2016	China (Asian)	360	368	199	141	20	539	181	217	135	16	569	167	PB	PCR-Taqman	Yes

*PCR-RFLP, polymerase chain reaction-restriction fragment length polymorphism; HWE, Hardy-Weinberg equilibrium*.

### Meta-analysis results

**Figure 2:**
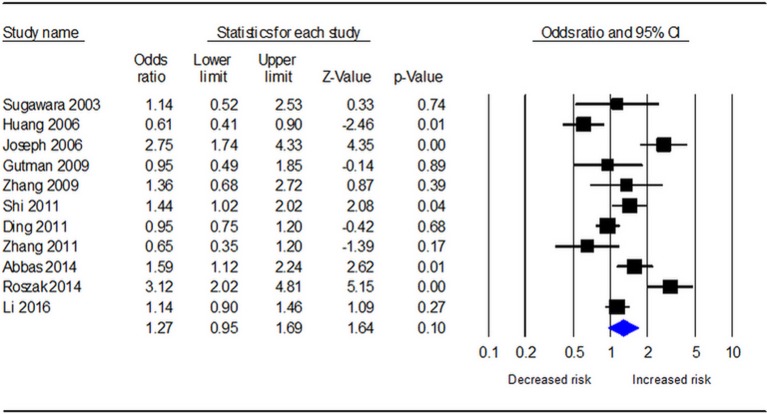
Forest plot for the relationship between *CYP1A1* Ile462Val polymorphism and cervical cancer risk under allele G vs. allele A genetic model.

**Figure 3:**
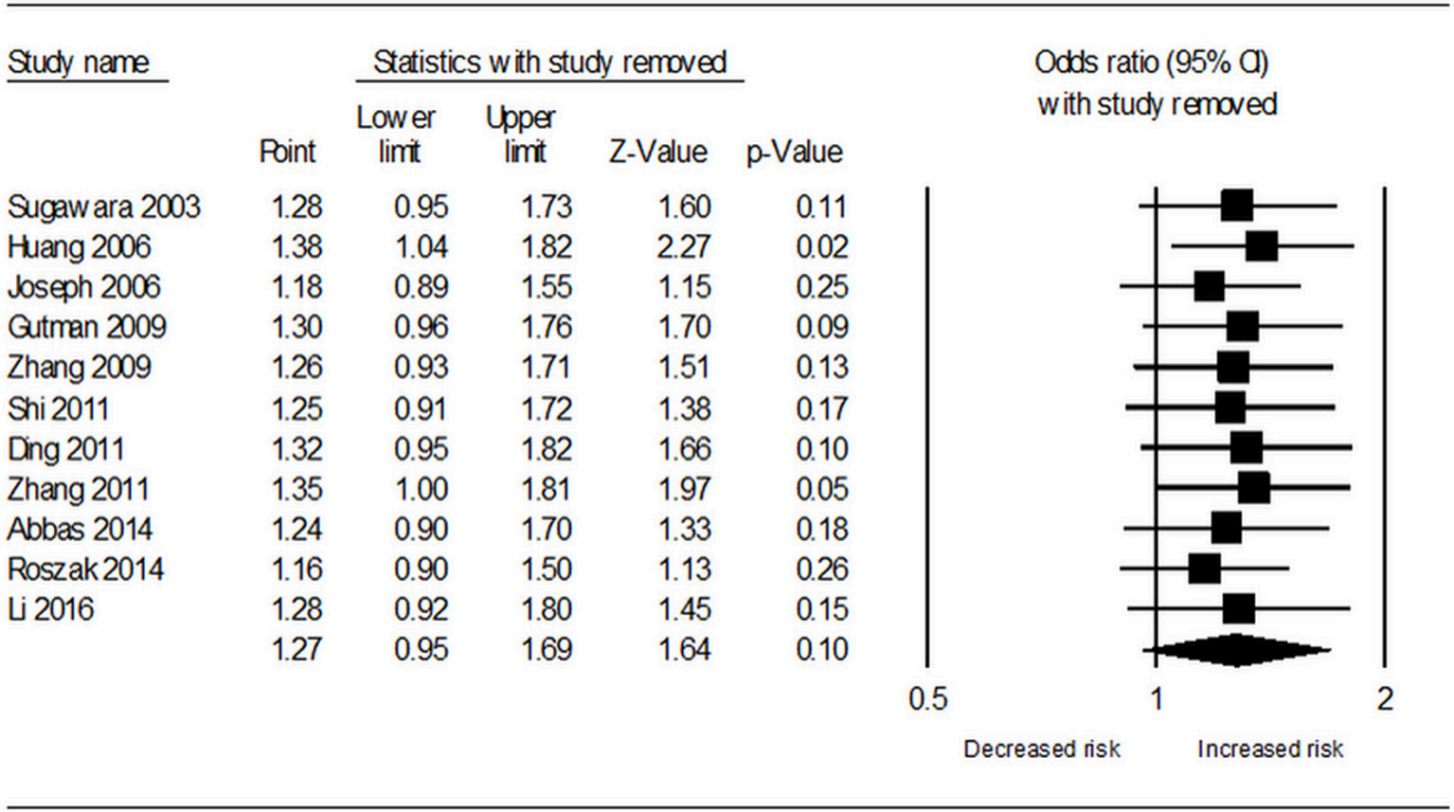
Sensitivity analysis results for removal of each included study under allele G vs. allele A genetic model.

**Figure 4:**
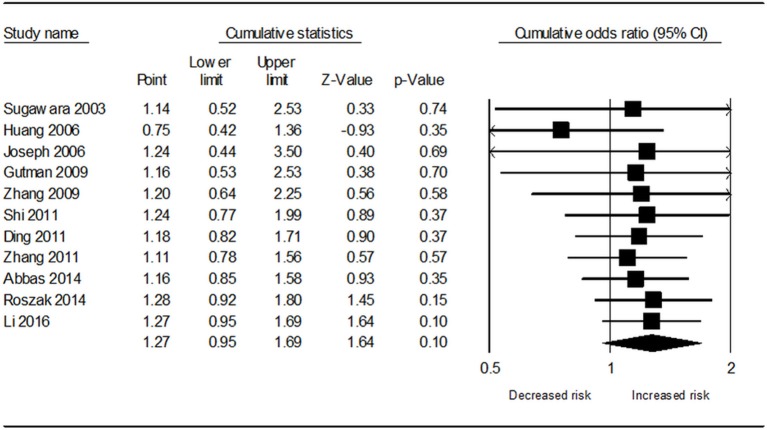
Cumulative meta-analysis of the association between *CYP1A1* Ile462Val polymorphism and cervical cancer risk under allele G vs. allele A genetic model.

Table 3 demonstrates the results of overall and subgroup analyses. Overall, the association between *CYP1A1* Ile462Val polymorphism and risk of cervical cancer was evaluated under five genetic models (G vs. A: OR = 1.27, 95% CI = 0.95–1.69, Figure 2; GG vs. AA: OR = 1.54, 95% CI = 0.87–2.74; GA vs. AA: OR = 1.27, 95% CI = 0.92–1.76; GA+GG vs. AA: OR = 1.25, 95% CI = 0.90–1.73; GG vs. AA+GA: OR = 1.31, 95% CI = 0.77–2.21), respectively. Sensitivity analysis indicated that the overall analysis was influenced only by Huang's study (Figure 3), which indicated that the results were robust. Moreover, cumulative meta-analysis was conducted by adding one study at a time in the order of publication year. The results showed that evidence of the effect of *CYP1A1* Ile462Val polymorphism on cervical cancer incidence was stable during the cumulative meta-analysis, but the CIs became increasing narrower (Figure 4). In total analysis, the association between *CYP1A1* Ile462Val polymorphism and cervical cancer incidence is borderline and may indicate a role of this variant.

**Table 3:** Meta-analysis results on the relationship of *CYP1A1* Ile462Val polymorphism with cervical cancer risk.

**Total and subgroups**	**Trails**	**G vs. A**		**GG vs. AA**		**GA vs. AA**		**(GA**+**GG) vs. AA**		**GG vs. (AA**+**GA)**	
		**OR (95%CI)**	***I*^2^(%)**	**OR (95%CI)**	***I*^2^(%)**	**OR (95%CI)**	***I*^2^(%)**	**OR (95%CI)**	***I*^2^(%)**	**OR (95%CI)**	***I*^2^(%)**
Total	11	1.27 (0.95–1.69)	81.67	1.54 (0.87–2.74)	54.99	1.27 (0.92–1.76)	72.99	1.25 (0.90–1.73)	75.05	1.31 (0.77–2.21)	52.36
**ETHNICITY**											
Asian	7	1.00 (0.79–1.26)	58.5	1.29 (0.72–2.30)	51.69	1.08 (0.66–1.75)	78.88	1.04 (0.66–1.64)	78.07	0.92 (0.69–1.22)	44.89
Caucasian	4	1.97 (1.24–3.13)	75.81	3.24 (1.24–8.46)	42.09	1.62 (1.25–2.10)	22.1	1.68 (1.16–2.43)	50.26	2.73 (1.05–7.10)	36.39
**SOURCE OF CONTROL**											
HB	3	0.76 (0.48–1.21)	50.72	0.92 (0.39–2.22)	35.54	0.85 (0.18–4.02)	89.44	0.77 (0.18–3.22)	89.18	0.80 (0.35–1.81)	0
PB	8	1.49 (1.10–2.02)	80.72	1.91 (0.94–3.87)	64.4	1.41 (1.20–1.67)	1.64	1.40 (1.19–1.64)	33.69	1.61 (0.79–3.28)	68.65
**HWE**											
No	5	1.11 (0.79–1.56)	76.56	1.79 (0.79–4.06)	57.43	1.22 (0.63–2.34)	85	1.17 (0.63–2.15)	84.31	1.38 (0.65–2.96)	57
Yes	6	1.42 (0.87–2.33)	84.29	1.23 (0.40–3.74)	63.86	1.36 (0.97–1.91)	50.6	1.34 (0.91–1.98)	64.31	1.24 (0.49–3.14)	52.09

In the subgroup analysis for ethnicity, no significant association was found in Asians under any genetic models, but significantly increased risk was observed in Caucasians under all contrasts (G vs. A: OR = 1.97, 95% CI = 1.24–3.13; GG vs. AA: OR = 3.24, 95% CI = 1.24–8.46; GA vs. AA: OR = 1.62, 95% CI = 1.25–2.10; GA+GG vs. AA: OR = 1.68, 95% CI = 1.16–2.43; GG vs. AA+GA: OR = 2.73, 95% CI = 1.05–7.10). After stratified analysis by source of controls, significant results were found in population-based controls (G vs. A: OR = 1.49, 95% CI = 1.10–2.02; GA vs. AA: OR = 1.41, 95% CI = 1.20–1.67; GA+GG vs. AA: OR = 1.40, 95% CI = 1.19–1.64). No significant association existed in the studies conforming to HWE or deviating from HWE under all five genetic models (Table 3).

### Publication bias

**Figure 5:**
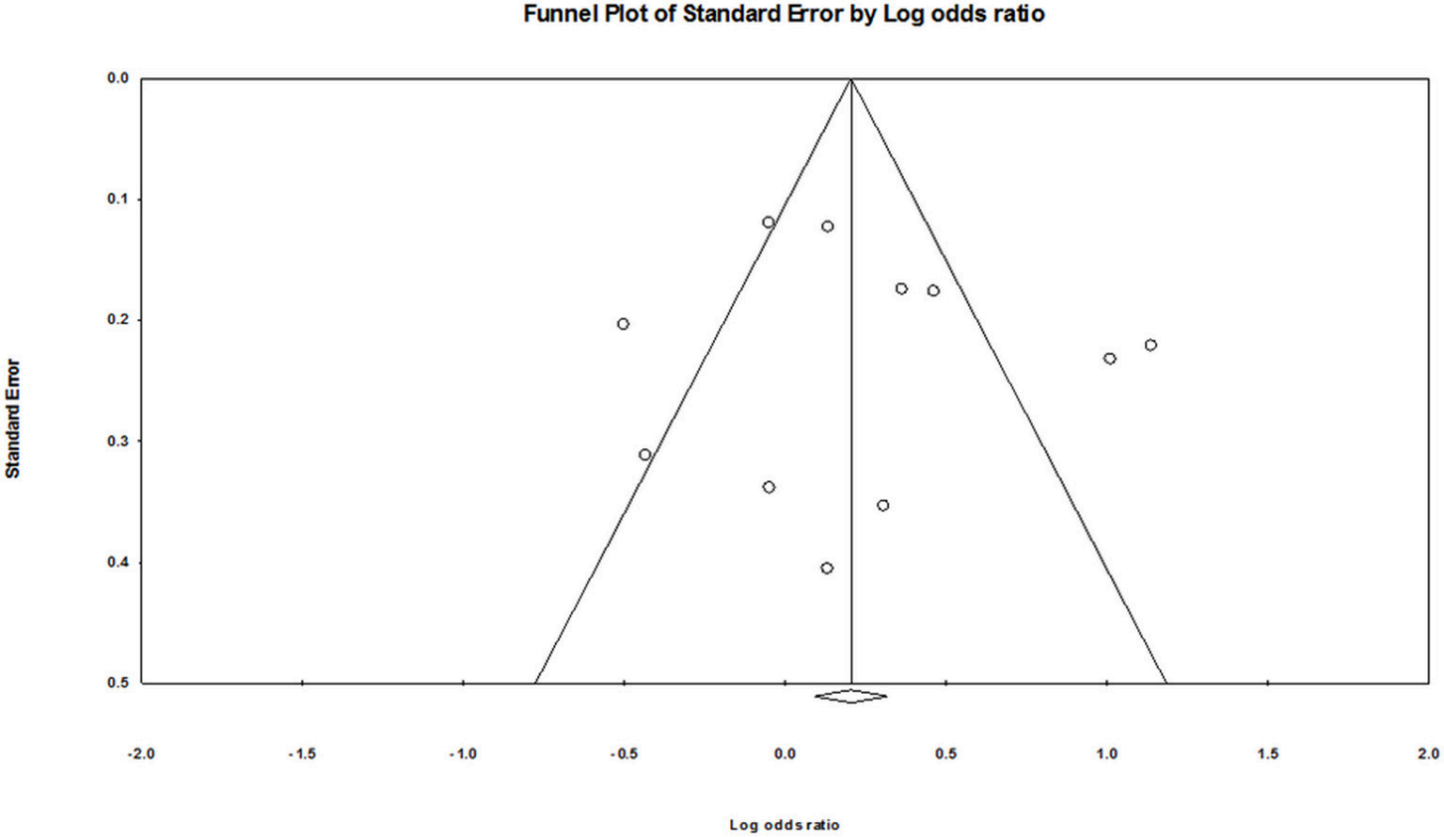
Funnel plot of publication bias for *CYP1A1* Ile462Val polymorphism.

As shown in Figure 5, no obvious publication bias was found. The Egger's test also showed no evidence of publication bias (G vs. A: *P* = 0.68; GG vs. AA: *P* = 0.57; GA vs. AA: *P* = 0.92; GA+GG vs. AA: *P* = 0.81; GG vs. AA+GA: *P* = 0.26).

## Discussion

Cervical cancer is the second most common cancer among women worldwide, frequently occurring in developing countries in particular (Forouzanfar et al., 2011; Denslow et al., 2012). To date, the pathogenesis of cervical cancer has not been identified utterly, but a few risk factors have been confirmed, including human papilloma virus (HPV) infection, smoking, multiparity and long duration of oral contraceptive use (Zeng et al., 2012; Patel et al., 2016). Currently, the complex interaction of environmental and genetic factors is also proposed to possibly conduce to the occurrence of cervical cancer.

Cytochrome P450 1A1 (*CYP1A1*) is a member of the CYP1 family and participates in the metabolic activation of structurally diverse xenobiotics and endobiotics (Rodriguez-Antona et al., 2010). So far, numerous studies have been conducted on the association between *CYP1A1* Ile462Val polymorphism and the susceptibility to cervical cancer, but the conclusions are not unanimous (Sugawara et al., 2003; Huang et al., 2006; Abbas et al., 2014). A few meta-analyses (Sergentanis et al., 2012; Yang et al., 2012; Wu et al., 2013; Qin et al., 2014; Wang et al., 2015) were also conducted to figure out the influence of *CYP1A1* Ile462Val polymorphism on cervical cancer susceptibility. However, due to different inclusive criteria and uneven sample sizes, these reports presented different conclusions. Although most of them indicated that *CYP1A1* Ile462Val polymorphism might be a risk factor for cervical cancer, the effects of the polymorphism on different ethnic groups were not fully clarified. Therefore, we conducted this cumulative meta-analysis to obtain accurate and up-to-date estimates of the association between the *CYP1A1* Ile462Val polymorphism and cervical cancer susceptibility.

In the current meta-analysis, we incorporated 11 case-control studies involving 1,932 patients and 2,039 healthy controls to statistically discuss this issue. According to our inclusion criteria, the controls clearly diagnosed as healthy subjects or tissues were included and those investigated the cervical dysplasia, cervical intraepithelial neoplasia (CIN), or cervical squamous intraepithelial lesions (CSIL) were excluded. Thus, the study by Taskiran et al. was not included in this meta-analysis (Taskiran et al., 2006). After data syntheses, we found a borderline correlation of *CYP1A1* Ile462Val polymorphism with the susceptibility to cervical cancer in total analysis. And the polymorphism significantly elevated the risk in Caucasian and population-based groups after subgroup analyses by ethnicity and source of control. Sensitivity analysis was conducted to evaluate the influence of individual study on overall analysis, which indicated that the overall results were robust. Furthermore, assessment of publication bias was confirmed by visual inspection of funnel plots and Egger's test, supporting that publication bias across the studies was negligible. Cumulative meta-analysis was also carried out to observe the change when with sample sizes were enlarged, and the CIs became increasing narrower. Recently, the relationships between some other polymorphisms and cervical cancer development were investigated, such as *CYP1A1* MspI (rs4646903), *COMT* (rs4680), and *CYP2E1* (rs3813867) polymorphisms. Importantly, a significant association between the MspI polymorphism and elevated cervical cancer risk was observed (Von et al., 2011; Matos et al., 2016). Studies suggest that Ile462Val polymorphism is in tight linkage disequilibrium with the MspI polymorphism, and these variant genotypes are associated with greater *CYP1A1* activity or inducibility (Crofts et al., 1994; Ng et al., 2005). Thus, the borderline association between Ile462Val polymorphism and cervical cancer risk cannot be excluded and may indicate a role of this variant, which warrants further functional studies.

Despite certain advantages, there still were some limitations to be addressed. To begin with, the sample sizes of subgroup analyses were relatively small, which might affect the final results. Second, since meta-analysis is a secondary analysis (Zeng et al., 2015), the possible impacts of gene-gene and gene-environment interactions with this polymorphism on cervical cancer susceptibility were not evaluated due to insufficient information from original papers. Third, only studies published in English or Chinese language were included, some unpublished due to negative findings or other reasons and in other languages might be missed.

In summary, our meta-analysis outcomes revealed a borderline relationship between *CYP1A1* Ile462Val polymorphism and cervical cancer risk in general population, and this polymorphism significantly increase the risk in Caucasian females. It provides an important theoretical basis to reveal the *CYP1A1* Ile462Val polymorphism and the biological mechanism of developing cervical cancer, which may be helpful to predict the occurrence of cervical cancer in Caucasian females. Importantly, special attention should be paid upon the design of future studies, such as selection of subjects and adjustment of interfering factors so as to uncover any underlying mechanisms and distinct patterns. In view of the above mentioned restrictions, these results should be applied with prudence and further verified by large-scale and well-designed studies with various ethnic groups.

## Author contributions

L-NW and X-QR designed this study; FW and Y-HJ searched databases and collected full-text papers; JL and CF extracted and analyzed data; L-NW, FW, and JL wrote the manuscript, X-QR reviewed the manuscript.

### Conflict of interest statement

The authors declare that the research was conducted in the absence of any commercial or financial relationships that could be construed as a potential conflict of interest.
